# Solving the Obstetrical Paradox: The FETAL Technique—A Step toward Noninvasive Evaluation of Fetal pH

**DOI:** 10.1155/2020/7801039

**Published:** 2020-02-08

**Authors:** Jacques Balayla, Guy Shrem

**Affiliations:** Department of Obstetrics and Gynecology, McGill University, Montreal, Quebec, Canada

## Abstract

Every year, about 85 percent of the approximately 5 million births in North America are evaluated with the electronic fetal monitoring (EFM). Clinicians use the EFM as a proxy to assess fetal oxygenation status, fetal well-being, and potential compromise. Despite the widespread use of this technology, neonatal hypoxia and acidosis continue to make up a high proportion of neonatal morbidity at term. Indeed, though the fetal heart rhythm is inextricably linked to fetal acid-base status, EFM has not been shown to reliably predict neonatal pH status nor has it reduced adverse maternal or neonatal outcomes. As a consequence, the high false-positive rate of EFM for predicting adverse neonatal outcomes has led to an increase in the rate of operative vaginal and cesarean delivery, with elevated rates of associated maternal and neonatal morbidity. This fact invariably leads to a paradox we have henceforth defined as the “obstetrical paradox.” Herein, we explore the potential solutions to this paradox and introduce a novel noninvasive technique to assess fetal acid-base status in utero known as the “FETAL technique” (Fourier Evaluation of Tracings and Acidosis in Labour). The FETAL technique, currently under investigation, applies the discrete Fourier transformation to EFM tracings to determine the spectral frequency distribution of the fetal heart rate. These specific frequency distributions correlate with specific umbilical pH values and may provide the missing link between fetal heat rate patterns and acid-base status at birth. As we work toward realizing the full potential benefits of EFM, finding the best assessment strategies to evaluate fetal pH in real time remains a key goal in obstetrics.

## 1. Introduction

### 1.1. Technological Advances in Obstetrics

Over the last several decades, technological advancements have brought forth an exponential increase in the understanding of basic biological and applied sciences and the interdisciplinary application of techniques across multiple areas of study [1].

Within the scope of obstetrics, a paradigm shift toward the use of noninvasive testing technologies beyond traditional ultrasonography has taken place. The prime examples include the detection of cell-free fetal DNA in maternal serum, which has allowed for the prenatal screening of fetal aneuploidies with sensitivities between 96.1 and 99.5% [2]. Similarly, Doppler studies of fetal circulation have revolutionized the management of fetal anemia and IUGR, leading to a major decline in perinatal mortality among patients with these conditions [3].

Nevertheless, over the last 30 years, little progress has been made in the screening and diagnosis of fetal acidosis. It has been well established that fetal acid-base status is inextricably linked to fetal heart rate patterns, a change in one leads to a change in the other. Though some headway was made with the widespread introduction of electronic fetal monitoring (EFM) [4], the presence of a marked pattern of late decelerations, a hallmark traditionally associated with poor neonatal outcomes, has been shown to predict acidosis only in half of cases [5]. Regrettably, EFM has not substantially changed the incidence of neonatal hypoxia, hypoxic ischemic encephalopathy, neonatal academia, cerebral palsy, or neurodevelopmental impairment [6]. Whereas some evidence suggests that intrapartum fetal monitoring is associated with a reduction in intrapartum death and neonatal seizures [6], a reduction in long-term neurologic disability—which is primarily related to oxygen status at birth—has not been demonstrated. Though EFM has moderate sensitivity, it has a low specificity for detecting fetal hypoxia/asphyxia [6]. As a consequence, the high false-positive rate of EFM for predicting adverse neonatal outcomes has in fact substantially enhanced the rate of operative vaginal and cesarean delivery, thereby increasing unnecessary maternal morbidity as well [6]. These findings are independent of whether EFM is used in low- or high-risk populations, including preterm pregnancies [6].

The reasons for the aforementioned findings are numerous, but a main concern outside of its face validity relates to the high interobserver and intraobserver variability, which occurs in the interpretation of EFM tracings [7]. Indeed, EFM was introduced into clinical practice without appropriate studies on its reliability (intra- and interobserver variability), validity (relationship of FHR patterns to fetal outcome), and causal relationship to outcome (ability of intervention to avoid metabolic acidemia). Though the development of a systematic and objective method of analysis and diagnosis in EFM was theorized to overcome these clinical limitations, several attempts to develop computerized and artificial intelligence (AI) systems for the quantitative and qualitative analysis of EFM have not demonstrated improvements in neonatal outcomes [7]. Indeed, in a meta-analysis our team conducted, we determined that relative to the use of clinical (visual) evaluation of the FHR, the use of AI did not change the incidence rates of neonatal acidosis, cord pH below 7.20, 5 min APGAR scores < 7, mode of delivery, NICU admission, neonatal seizures, or perinatal death. With regard to the degrees of interrater reliability, we found a weighed mean Cohen's kappa of 0.49 (0.32–0.66), which indicates moderate agreement between expert observers and computerized systems [8]. It is thus understood that current EFM interpretation may not be a reliable predictor of neonatal pH status, and as such, it has failed as a public health screening program.

As we work toward realizing the full potential benefits of electronic fetal monitoring, finding the best assessment strategies to evaluate fetal pH remains a key goal in obstetrics, as it will inevitably lead to a reduction in interventions. In the meantime, we are forced to face the principal limitation that assessing fetal acid-base status holds—namely, that it can only be carried out using invasive methods.

### 1.2. Assessment of Fetal and Neonatal pH Status

Fetal acid-base balance can be assessed using diagnostic techniques at three main clinical set points during pregnancy: (1) during antepartum, by percutaneous umbilical cord blood sampling (PUBS); (2) during the intrapartum period, by fetal scalp blood sampling; and (3) immediately after birth, by umbilical cord blood sampling. Unfortunately, not all providers or all centers are equipped with the technical expertise to carry these out and only fetal scalp blood sampling provides a way to estimate fetal acid-base status when it matters most—namely, during labour.

The etiology and diagnosis of neonatal hypoxic ischemic encephalopathy (HIE) reveals the complexity of prenatal diagnosis of this complication. Indeed, the diagnosis is typically based on a combination of medical history, physical and neurological exams, laboratory results, and neuroimaging. However, the insult leading to HIE can take place at any time during pregnancy, with particular predilection for labour. This is why the real-time evaluation of fetal pH is crucial during labor: it will allow us to determine the exact timing of injury should it occur and provide a true critical window for intervention that may benefit the newborn's acid-base status.

## 2. The Obstetrical Paradox

The aforementioned methods to determine fetal pH are invasive and complex and may put the fetus at substantial risk. Other than the biophysical profile (BPP), the only current noninvasive tool at our disposal during labour, which is not diagnostic, is the EFM. Since the EFM has not been shown to reliably predict neonatal pH status or reduce adverse outcomes, one fundamental conundrum, hereby termed the *obstetrical paradox*, arises: if the FHR is inextricably linked to fetal acid-base status and the monitoring reflects the fetal heart rhythm over time, why are neonatal outcomes not improved when tracing changes are detected and acted upon?

**Figure 1:**
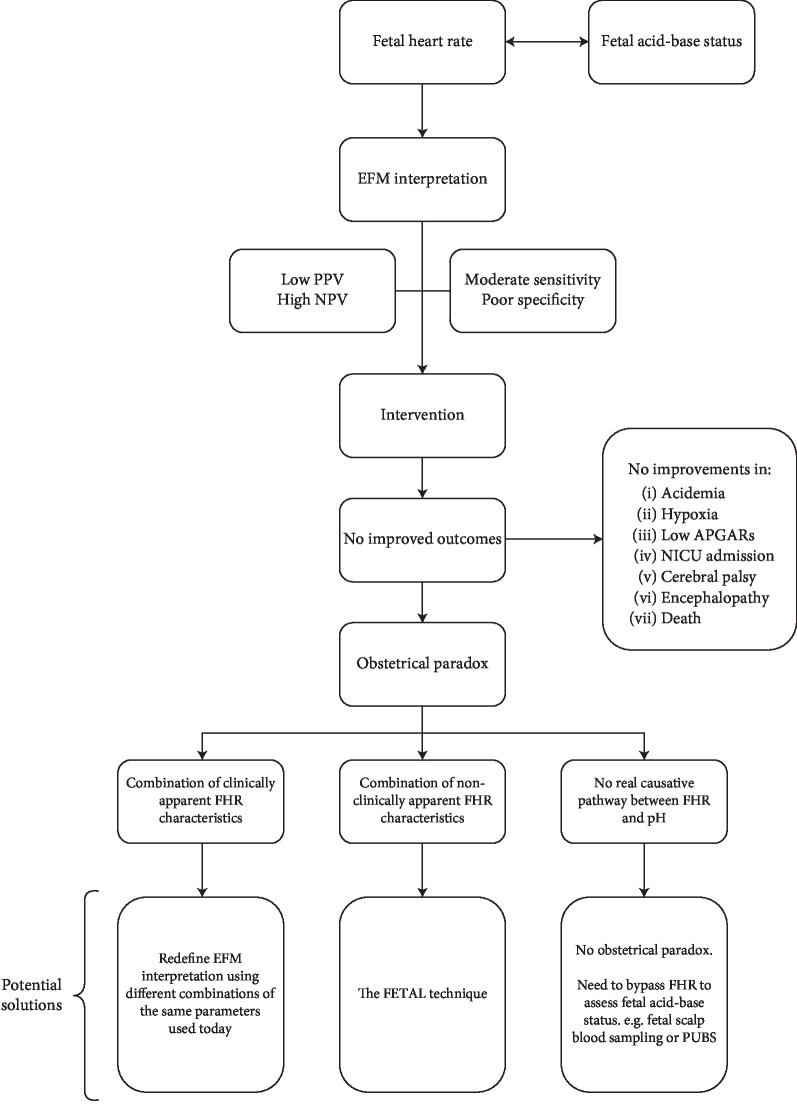
The obstetrical paradox.

Outside of human error and delays in intervention, a potential solution to the obstetrical paradox (Figure 1) may be related to the possibility that the modern interpretation of EFM is incomplete—that other visible tracing characteristics we do not presently account for are actually predictive of fetal pH and lead to better outcomes when acted upon. A second possibility is that perhaps there is merely an association and not a causal pathway between changes in pH and EFM fluctuations. Such explanation would thus imply that there is no direct relationship between the fetal heart rhythm and fetal pH, and thus, there is no paradox at all. Finally, it may be the case that the key to predicting fetal pH lies embedded within EFM tracings but beyond their traditional clinical interpretation as set by the United States National Institute of Child Health and Human Development (NICHD) [9].

### 2.1. Potential Solutions to the Obstetrical Paradox

The most valid way to *screen* for fetal pH is unknown. However, we can reliably hypothesize that it either lies within the FHR or it does not. Furthermore, we can reliably affirm that the current method to assess fetal oxygenation status via the FHR, as proposed by the NICHD [9], fails to reliably and consistently predict fetal compromise. Therein lies the core of the obstetrical paradox. Given the aforementioned considerations, two potential scenarios arise in the context of solving the said paradox. Herein, we explore each of these scenarios and discuss potential insights that may enhance this debate.

#### 2.1.1. Scenario #1: Redefine the Current FHR Interpretation Using Traditional Parameters

The current guidelines for EFM classification use a combination of baseline heart rate, variability, accelerations, and decelerations to determine whether an FHR pattern is to be classified as normal, abnormal, or atypical (different organizations use different terminologies to classify these patterns). There are strict criteria that use specific characteristics of these variables to establish fetal well-being or compromise. As we have established above, this classification is insufficient to reliably predict fetal compromise, with large numbers of false-positive interpretations leading to unnecessary interventions and no improvements in fetal outcomes. One potential solution to such dilemma is therefore that different criteria be used to interpret the FHR. As a theoretical example, perhaps a more sensitive and specific predictor of fetal compromise is not simply the presence of decelerations but rather the ratio of accelerations to decelerations? Likewise, should there be a weighted consideration of these parameters whereby variability plays a more prominent role in the consideration of fetal compromise? Though it is conceivable that a different theoretical combination of these parameters may improve the sensitivity and specificity of the FHR, as it stands, there is no literature studying different interpretations of the FHR using these traditional parameters.

#### 2.1.2. Scenario #2: Using Nonclinically Apparent Features of the FHR to Enhance Specificity

It is conceivable that though the aforementioned parameters are readily visualized on EFM tracings, they may not predict fetal acid-base status in real time. Instead, perhaps other characteristics that are not clinically apparent may provide insight into fetal well-being. In the authors' view, this is a more likely assumption. To explore this hypothesis, we have developed the FETAL technique.

## 3. The FETAL Technique

The FETAL technique, which stands for the “Fourier Evaluation of Tracings and Acidosis in Labour,” is an innovative method, which applies the discrete Fourier transformation to EFM tracings in order to determine the spectral frequency and power distribution of the FHR. The Fourier transformation is a mathematical tool, which converts a time-dependent waveform signal, such as the FHR, into a frequency and power spectrum domain of fundamental sinusoidal waves [10]. Operations performed in a frequency domain elicit properties of the original function that are not immediately apparent on visual inspection [10]. By applying the FETAL technique clinically, specific frequency distribution patterns of the FHR can be correlated with specific umbilical pH values in the newborn. Though not yet tested clinically, these frequency distributions in animal models have been theorized to provide the missing link between fetal heat rate patterns and acid-base status at birth [11]. Simply put, by decomposing the EFM signal in real time into its primordial components using the Fourier transform, the FETAL technique will seek to establish a sensitive, specific, and universal approach to reduce uncertainty in the interpretation of the FHR. Whether AI assistance in this task will enhance the sensitivity of EFM interpretation and reduce adverse outcomes remains to be observed. We have enumerated the numerous implications of a successful FETAL technique in Table 1.

## 4. Physiological Basis for the Application of the FETAL Technique

Heart rate variability investigations demonstrated that changes in FHR variability precede changes in actual heart rate in cases of intrauterine asphyxia [12]. Indeed, the heart rate depends on the sinus node's intrinsic rate and sympathetic-parasympathetic nervous tone balance, which themselves are directly dependent on oxygenation status [13]. Power spectral analysis of FHR variability has shown that sympathetic and parasympathetic nervous activities make frequency-specific contributions to the FHR power spectrum [11, 13]. Specifically in the nonanomalous term fetus, heart rate variability estimated by the high-frequency (HF) bands between 0.15 and 0.45 Hz reflects FHR control by the parasympathetic tone and the low-frequency (LF) bands between 0.03 and 0.15 Hz reflects the sympathetic tone [11, 13]. In clinical terms, moderate baseline variability reflects the oxygenation of the central nervous system and reliably predicts the absence of damaging degrees of hypoxia-induced metabolic acidemia at the time it is observed. However, the converse is not true: minimal or absent variability alone is a poor predictor of acidemia or hypoxic injury at the time it is observed. Studies of heart rate fluctuation based on frequency analysis have been carried out in animal models [11, 14]. However, their clinical application in humans has not been studied as of yet. These studies have found that the shift of autonomic balance is related to the redistribution of the power between the LF and HF bands and that normalized power units or LF/HF are effective methods of determining the shift of autonomic balance—the mechanism presumed to be at the helm of fetal acid-base status. By studying specific low- and high-frequency patterns of Fourier transformed domains, we are in the process of setting up a clinical trial to study the FETAL technique in the clinical setting, as we seek to determine whether fetal acid-base status evaluations can be made noninvasively and in real time.

Compounding the limitations of the EFM and adding to the cesarean epidemic, physicians are under great pressure because of medicolegal concerns as well. This situation increases the rate of unnecessary interventions like cesarean section, operative delivery, and iatrogenic preterm delivery. As we continuously improve the quality of obstetrical care we provide, areas of potential substantial impact, where good evidence-based gaps exist, will need to be addressed primarily. Developing noninvasive methods to enhance the sensitivity and specificity of the EFM to evaluate fetal acid-base status is one such area, whose potential implications of reducing rates of neonatal asphyxia while lowering the cesarean and assisted vaginal delivery rates are paradigm-altering.

## Figures and Tables

**Table 1:** Summary overview of the implications of a successful FETAL technique.

(1) Identification of true fetal distress (a) Allows for adequate resuscitation or expedited delivery where appropriate (b) Reduces rates of NICU admission (2) Reduction in the rate of cesarean delivery for suspected fetal distress (a) Reduces future rates of repeat cesarean delivery (b) Reduces risk of abnormal placentation and its associated complications (c) Reduces maternal morbidity and mortality (3) Reduction in the rate of assisted vaginal delivery for suspected fetal distress (a) Reduces maternal morbidity (b) Reduces neonatal morbidity (4) Lower rates of intervention during labour (a) Enhances chances of completing a spontaneous vaginal delivery (5) Reduced rates of parental and provider psychological distress (a) Enhances therapeutic relationship between patient, family, and provider (b) Enhances chances of completing a spontaneous vaginal delivery (6) Forensic analysis of tracings (a) Allows for education of providers (b) Identifies key window in labour where fetal distress is present
